# Thirty-eight-year trends of educational differences in smoking in Finland

**DOI:** 10.1007/s00038-019-01228-x

**Published:** 2019-03-25

**Authors:** Otto Ruokolainen, Antero Heloma, Pekka Jousilahti, Jouni Lahti, Oona Pentala-Nikulainen, Ossi Rahkonen, Pekka Puska

**Affiliations:** 1 National Institute for Health and Welfare, Po Box 30, 00271 Helsinki, Finland; 2 Department of Public Health, University of Helsinki, Helsinki, Finland; 3 Statistics Finland, Helsinki, Finland

**Keywords:** Smoking, Socio-economic position, Inequalities, Population-based survey, Price, Trends

## Abstract

**Objectives:**

Smoking is declining, but it is unevenly distributed among population groups. Our aim was to examine the socio-economic differences in smoking during 1978–2016 in Finland, a country with a history of strict tobacco control policy.

**Methods:**

Annual population-based random sample data of 25–64-year-olds from 1978 to 2016 (*N* = 104,315) were used. Response rate varied between 84 and 40%. In addition to logistic regression analysis, absolute and relative educational differences in smoking were examined.

**Results:**

Smoking was more prevalent among the less educated but declined in all educational groups during the study period. Both absolute and relative differences in smoking between the less and highly educated were larger at the end of the study period than at the beginning. Cigarette price seemed to have a larger effect on the smoking among the less educated.

**Conclusions:**

Socio-economic differences in smoking among the Finnish adult population have increased since the 1970s until 2016. Further actions are needed, especially focusing on lower socio-economic positions, to tackle inequalities in health. They should include support for smoking cessation and larger cigarette tax increases.

**Electronic supplementary material:**

The online version of this article (10.1007/s00038-019-01228-x) contains supplementary material, which is available to authorized users.

## Introduction

The detrimental effects of smoking on health are well known and reported (USDHHS 2014). Smoking has declined in Europe since the 1980s, but it is differently distributed among the population (Ng et al. 2014; Graham 1996; European Commission 2003, 2017). Men and lower socio-economic groups generally smoke more than women and the higher socio-economic groups, and the differences between socio-economic groups seem to have increased (Schaap et al. 2008; Hoebel et al. 2018; Lahelma et al. 2016; Alves et al. 2015). Thus, smoking is a significant factor creating and sustaining inequalities in health among population groups (Kulik et al. 2013, 2014).

A central aim of Finnish health policy, in addition to improving public health, is to reduce inequalities in health (Melkas 2013). In tobacco control, legislation has a history of four decades, as the first Tobacco Control Act (TCA) was implemented in 1977 (Patja 2014). Smoking restrictions in public places, a ban on advertising, and sales to minors were the main components of the first TCA. Since then, the TCA has been tightened several times, for example to include smoking bans in workplaces (1995) and restaurants (2003, fully implemented in 2007) and point-of-sale display bans (2012). In 2010, Finland took a step forward at eradicating inequalities when the objective of the TCA was stated as to end the use of tobacco products instead of just reducing it (the so-called endgame). The target year was set to 2030 in 2016 and to also include “other nicotine-containing products that are toxic to humans and cause addiction” (medicinal nicotine replacement therapy excluded) (Finlex 2016). Even though several countries have adopted the endgame as a governmental strategy, Finland is the only country where the endgame is explicitly stated as the objective of the TCA.

It is proposed that both price and non-price tobacco control policies implemented in nine European countries in 1990–2007, including Finland, have helped to reduce the prevalence of smoking especially in lower socio-economic groups. Still, inequalities in smoking have widened during this time (Hu et al. 2017). On the EU level in the 2000s, implemented tobacco control policies have promoted smoking cessation and decreased the intensity of smoking more among the highly educated than among the less educated (Bosdriesz et al. 2016).

The price of tobacco is also highly influential in smoking (Thomas et al. 2008; Yeh et al. 2017). In Finland, after a long period with no raises, the nominal price of cigarettes increased by 61% in 2008–2016 (Tobacco Statistics 2017). According to the Tobacco Control Scale 2016, the price of tobacco products in Finland is still far from the leading UK (Joossens and Raw 2017).

In sum, clear socio-economic differences in smoking have been found in earlier studies (Lahelma et al. 2016; Hu et al. 2017) and studies show that socio-economic differences in smoking are not decreasing but persisting or even increasing in recent years (Hoebel et al. 2018; Sandoval et al. 2018). In Finland, since the late 1970s until recent years, these differences are unknown. The aim of this study is to describe the socio-economic differences in smoking and to examine whether these differences have widened. To explore this, two research questions are proposed: How did smoking prevalence change since 1978 to 2016 among different educational groups? Have the absolute and relative differences in smoking between educational groups increased since 1978?

## Methods

Nationwide Health Behaviour and Health among the Finnish Adult Population data 1978–2014 were used. It is an annual postal survey with 5000 15–64-year-olds randomly drawn from the National Population Register. The 2016 data come from the Regional Health and Well-being Study, an annual postal and web survey with 5000 respondents aged 20 and over, randomly drawn from the National Population Register. The response rate varied from 84% in 1978–1979 to 40% in 2016. Data for 2015 were not available. We examined 25–64-year-olds as the educational level might still be in the process for younger respondents. Our final data consisted of 104,315 respondents. The protocol of the surveys has been accepted by the Institutional Review Board of National Institute for Health and Welfare.

**Fig. 1:**
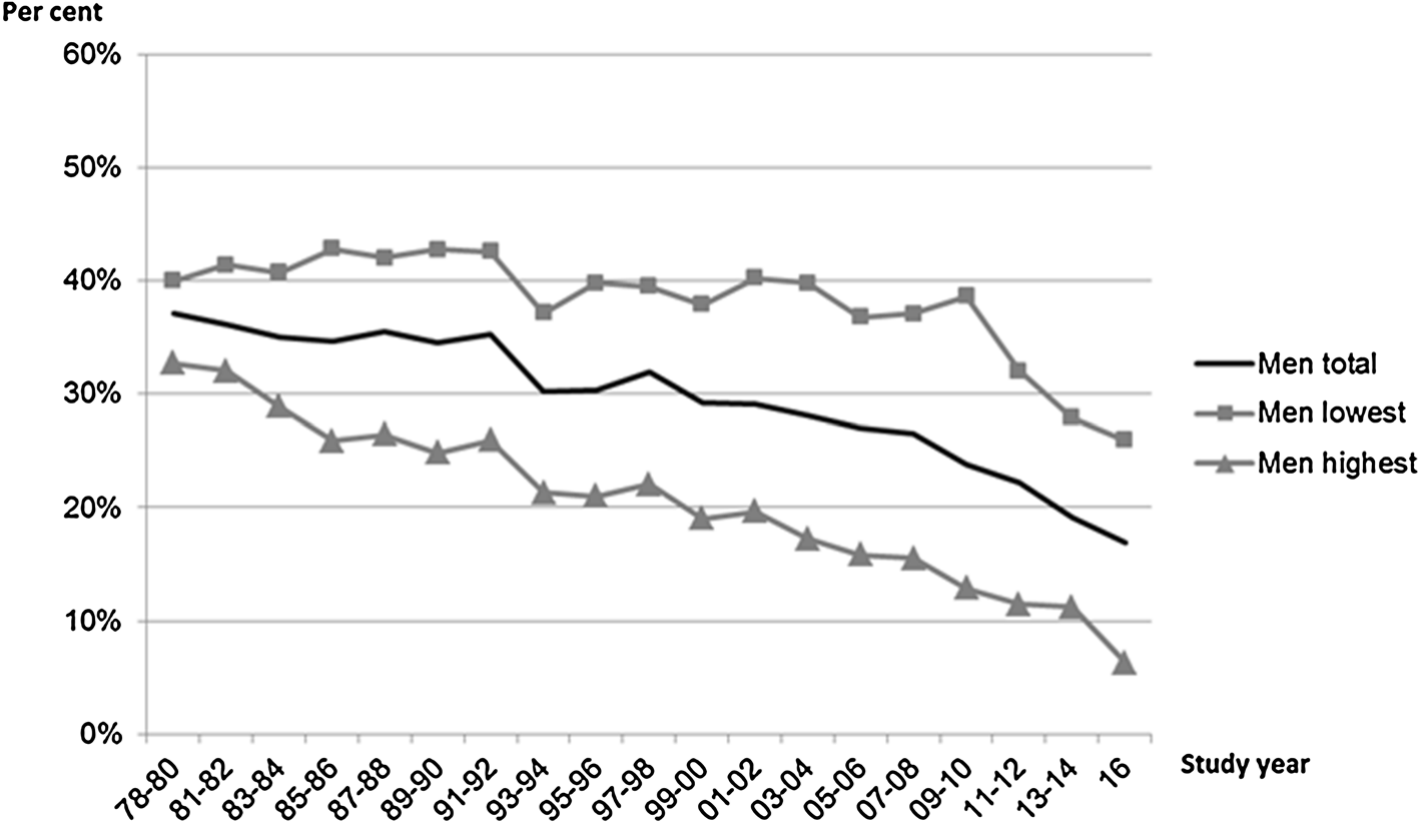
Daily smoking by education, men, 25–64 years, age adjusted. Finland, 1978–2016, Health Behaviour and Health among the Finnish Adult Population/Regional Health and Well-being Study

**Fig. 2:**
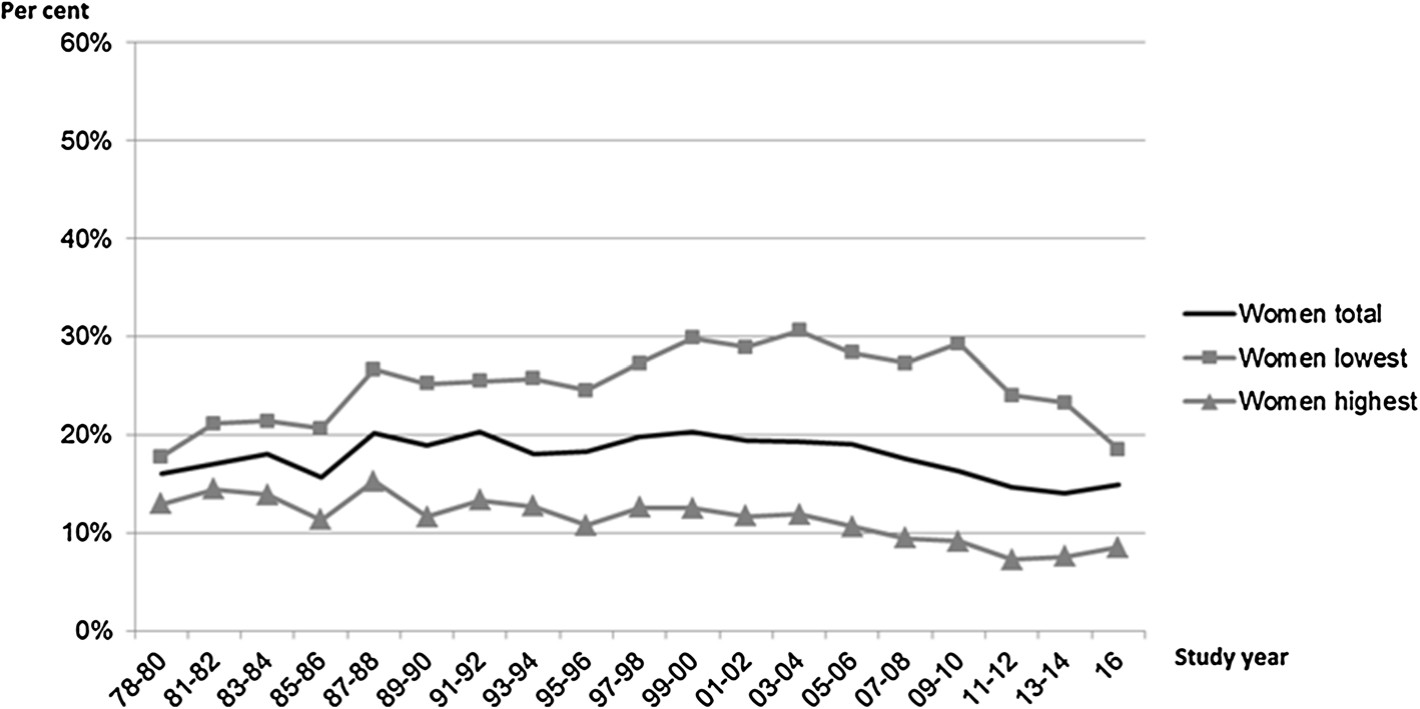
Daily smoking by education, women, 25–64 years, age adjusted. Finland, 1978–2016, Health Behaviour and Health among the Finnish Adult Population/Regional Health and Well-being Study

In order to match the age–sex distribution of the total Finnish adult population in the census register, post-stratification weights using the total Finnish adult population aged 25–64 years as the reference population were computed. Distributions for 10-year age groups (25–34, 35–44, 45–54, and 55–64) and alternatively 20-year age groups (25–44 and 45–64) according to sex and education (tertiles) were used to compute weights for each case. For the total prevalence estimates (solid black lines shown in Figs. 1 and 2), only age and sex were used to compute post-stratification weights. Weights (*pweight*) were used in all analyses if not noted otherwise.

### Variables

Smoking status was defined with three and since 1996 four questions following the World Health Organization’s recommendations (World Health Organization 1998): ‘Have you ever smoked’, ‘Have you ever smoked daily at least 1 year/How many years?’, ‘When was the last time you smoked?’, and since 1996, ‘Have you ever smoked at least 100 times (cigarettes, cigars, pipes)?’. The final variable included four categories ‘Daily smoker’, ‘Occasional smoker’, ‘Former smoker’, and ‘Never smoker’ (see Online Resource A for the determination of smoking status). Incomplete data (~ 5%) were omitted. Binary daily smoking was used as the outcome variable in all analyses.

The educational structure has changed during the study period. In 1970, the proportion with the highest educational level was 9% and 28% in 2010 among the Finnish population. Similarly, the proportion with the lowest educational level has decreased from 75% (1970) to 29% (2010) (Statistics Finland 2018). Thus, relative education was used as an indicator of socio-economic position. For each survey year, the self-reported number of school years was stratified according to tertile cut points (‘less’, ‘middle’, and ‘highly educated’), taking into account the sex of the respondent and the year of the response. For analyses, we compared the less educated to the highly educated. Two successive survey years were pooled together to strengthen the statistical power of the analyses. The first 3 years were pooled together and the last survey year was separate in the analyses.

### Statistical analyses

To answer the research question ‘How did smoking prevalence change since 1978 to 2016 among different educational groups?’, the following steps were taken. First, age-adjusted daily smoking among educational groups was graphically observed (Figs. 1, 2). Then, to examine the trends in smoking in socio-economic groups, the linear effect of time points on daily smoking was tested with logistic regression models stratified by sex and educational group (see Table 2). In this, survey year was coded as a continuous variable: for example 1981–1982 was coded as 0.000, 1983–1984 as 0.056, 1985–1986 as 0.111,…, and 2016 as 1.000 (Hoebel et al. 2018). These analyses were restricted to the years 1981–2016 to maintain comparability between the models both excluding and including the real price index (see below the description for the real price index). Same kind of analysis was performed from 2001 onwards based on the visual examination of Figs. 1 and 2 (see Table 2, Panel B). Stratification by age groups 25–45 and 45–64 was additionally conducted. These age groups were used to classify respondents as ‘younger’ and ‘older’ participants, meanwhile maximizing the number of daily smokers in each group for statistical tests. A squared term of the survey year for the trend in time was included for the less educated women (excluding the trend from 2001 onwards, see Table 2, Panel B) to account for the quadratic trend shown in Fig. 2 and Online Resource C (only among the younger age group).

To answer the research question ‘Have the absolute and relative differences in smoking between educational groups increased since 1978?’, absolute and relative group differences in smoking were examined using the slope index of inequality (SII) and the relative index of inequality (RII) (Regidor 2004). These summary indices are regression-based estimates that measure hierarchical group differences intended to be used in parallel to get a more thorough picture of the phenomenon. Recent studies have used the same methods for comparing smoking between socio-economic groups (Hoebel et al. 2018; Lahelma et al. 2016; Ernstsen et al. 2012). We followed the method used by Ernstsen et al. (2012) for computing SII and RII models. Educational groups by sex and survey year were given a decreasing value from 1.000 to 0.000, according to the age-adjusted prevalence of the relative educational level. The calculated measure (ridit score) was then used as an independent variable in an age-adjusted generalized least-squares model. The analyses were stratified by sex and survey year. For testing the trend in SII/RII over time between socio-economic groups, survey year and interaction variable survey year*ridit score were included in the model. Post-stratification weights were not used for SII/RII analyses since the calculated ridit score was already age-adjusted according to sex and educational level.

Real price index (= cigarette price index/consumer price index) was used as a covariate in the logistic models as well as SII and RII calculations to account for the effect of price changes on smoking. The available data for 1981–2016 were obtained from Statistics Finland. The mean value of annual averages for two subsequent years to match the year variable was calculated. For 2016, we used the mean value for 2015 and 2016. Because the price index was not available for 1978–1980, analyses including the price index only account for the year 1981 onwards. Complete cases were available for all analyses. SPSS 25 and StataSE 15 were used for data management and analyses, applying 95% confidence level.

## Results

Daily smoking among men declined from 37% (1978–80) to 17% (2016). Daily smoking among women first slightly increased from 16% (1978–80) to 18–20% (1987–2006) and after that declined to 15% (Table 1, Figs. 1, 2). Smoking among the less educated was more prevalent during the whole study period compared with the highly educated among both sexes. Smoking decreased among both less and highly educated men. Among the less educated women, smoking first increased, but started to decrease in the early 2000s. Smoking among highly educated women peaked in the late 1980s and then gradually decreased. Decreasing trends for daily smoking over time for men and women among different educational groups were statistically significant (Table 2, Panel A). Adjustment for the real price index explained the association only among the less educated men. Based on the visual examination of Figs. 1 and 2, the trend seemed to change especially for the less educated women in the early 2000s. Thus, additional trend analyses were performed starting from 2001 until 2016. A declining trend was observed in both less and highly educated women and men (Table 2, Panel B). The trend remained statistically significant only among highly educated men and highly educated women after the adjustment for the real price index.

**Table 1:** Age-adjusted prevalence of smoking status of participants by sex, 25–64-year-olds, Finland 1978–2016. Health Behaviour and Health among the Finnish Adult Population/Regional Health and Well-being Study

Year	Men					Women				
	*N* ^a^	Daily smoker (%)	Occasional smoker (%)	Former smoker (%)	Never smoker (%)	*N* ^a^	Daily smoker (%)	Occasional smoker (%)	Former smoker (%)	Never smoker (%)
1978–1980	5574	37	5	29	29	5381	16	4	10	70
1981–1982	3311	36	5	26	33	2956	17	4	11	68
1983–1984	2868	35	6	26	33	3096	18	5	11	66
1985–1986	2597	35	6	27	32	3011	16	5	13	66
1987–1988	2787	36	6	23	36	3191	20	5	13	62
1989–1990	2807	35	6	24	35	3106	19	6	12	64
1991–1992	2680	35	6	23	35	3119	20	4	14	61
1993–1994	2494	30	7	26	37	2842	18	5	13	64
1995–1996	2629	30	7	27	36	3058	18	5	16	60
1997–1998	2614	32	6	26	36	2918	20	5	16	59
1999–2000	2449	29	6	26	39	2942	20	5	16	59
2001–2002	2444	29	6	26	38	2854	19	5	18	58
2003–2004	2421	28	7	26	40	2918	19	5	18	57
2005–2006	2417	27	7	25	41	2874	19	6	19	56
2007–2008	2316	27	8	26	40	2969	18	5	21	56
2009–2010	2083	24	8	23	45	2672	16	6	21	58
2011–2012	1940	22	8	26	44	2547	15	6	22	58
2013–2014	1868	19	8	24	49	2446	14	5	20	60
2016	466	17	8	29	46	650	15	4	23	57

^a^N from weighted data

**Table 2:** Logistic regression models for trend for daily smoking in 1981–2016 (A) and in 2001–2016 (B) by sex and educational level. Age-adjusted odds ratios and their 95% confidence intervals, 25–64-year-olds. Finland, 1981–2016, Health Behaviour and Health among the Finnish Adult Population/Regional Health and Well-being Study

	A. Trend for daily smoking, 1981–2016		B. Trend for daily smoking, 2001–2016	
	Lowest	Highest	Lowest	Highest
Men	0.65 (0.57, 0.74)	0.26 (0.22, 0.30)	0.25 (0.15, 0.42)	0.12 (0.06, 0.22)
Men^a^	0.75 (0.48, 1.17)	0.21 (0.12, 0.36)	0.36 (0.11, 1.16)	0.17 (0.04, 0.75)
Women	0.19 (0.11, 0.32)	0.52 (0.44, 0.62)	0.35 (0.21, 0.56)	0.21 (0.10, 0.44)
Women^a^	0.15 (0.09, 0.28)	0.36 (0.19, 0.67)	0.53 (0.17, 1.62)	0.16 (0.03, 0.77)

^a^Adjusted additionally for the real price index

Table 3 shows the results for absolute (SII) and relative (RII) differences in smoking throughout the study period. There was some fluctuation in estimated differences from year to year, especially observable in the 2000s. Broadly, both of the estimates increased over time, indicating widening absolute and relative differences in smoking between educational groups. Statistically significant trends remained also after adjusting for the real price index (all models *p* < 0.001 for the variable survey year*ridit score).

**Table 3:** Slope index of inequality (SII) and relative index of inequality (RII) of daily smoking by sex, 25–64-year-olds. Finland, 1978–2016, Health Behaviour and Health among the Finnish Adult Population/Regional Health and Well-being Study

Year	Men		Women	
	SII (95% CI)	RII (95% CI)	SII (95% CI)	RII (95% CI)
1978–1980	0.11 (0.07, 0.16)	1.37 (1.21, 1.55)	0.05 (0.01, 0.08)	1.63 (1.31, 2.03)
1981–1982	0.14 (0.09, 0.20)	1.53 (1.29, 1.80)	0.06 (0.01, 0.10)	1.86 (1.39, 2.49)
1983–1984	0.18 (0.12, 0.25)	1.69 (1.41, 2.03)	0.08 (0.03, 0.13)	1.97 (1.50, 2.59)
1985–1986	0.26 (0.20, 0.33)	2.15 (1.77, 2.61)	0.10 (0.05, 0.14)	2.60 (1.91, 3.53)
1987–1988	0.25 (0.18, 0.31)	1.96 (1.63, 2.35)	0.15 (0.10, 0.20)	2.54 (1.97, 3.28)
1989–1990	0.28 (0.21, 0.34)	2.23 (1.84, 2.68)	0.18 (0.13, 0.23)	3.04 (2.30, 4.00)
1991–1992	0.26 (0.20, 0.33)	2.14 (1.77, 2.58)	0.15 (0.09, 0.20)	2.50 (1.93, 3.23)
1993–1994	0.24 (0.17, 0.31)	2.19 (1.76, 2.73)	0.16 (0.11, 0.21)	2.98 (2.22, 3.99)
1995–1996	0.27 (0.21, 0.34)	2.60 (2.09, 3.23)	0.19 (0.14, 0.24)	3.08 (2.32, 4.09)
1997–1998	0.26 (0.19, 0.32)	2.28 (1.85, 2.82)	0.20 (0.15, 0.25)	3.06 (2.32, 4.03)
1999–2000	0.26 (0.20, 0.33)	2.44 (1.94, 3.06)	0.24 (0.19, 0.29)	3.72 (2.83, 4.90)
2001–2002	0.29 (0.23, 0.36)	2.91 (2.30, 3.68)	0.24 (0.19, 0.29)	3.83 (2.87, 5.12)
2003–2004	0.32 (0.25, 0.38)	3.21 (2.52, 4.08)	0.24 (0.19, 0.29)	4.07 (3.05, 5.42)
2005–2006	0.29 (0.23, 0.36)	3.00 (2.34, 3.85)	0.25 (0.20, 0.30)	3.96 (2.96, 5.30)
2007–2008	0.31 (0.24, 0.37)	3.32 (2.55, 4.31)	0.25 (0.20, 0.30)	4.45 (3.30, 6.00)
2009–2010	0.35 (0.28, 0.41)	4.62 (3.43, 6.21)	0.24 (0.20, 0.29)	5.87 (4.17, 8.26)
2011–2012	0.32 (0.26, 0.39)	4.02 (2.94, 5.50)	0.24 (0.19, 0.29)	5.40 (3.75, 7.77)
2013–2014	0.26 (0.19, 0.32)	3.66 (2.59, 5.18)	0.21 (0.16, 0.26)	5.05 (3.45, 7.38)
2016	0.30 (0.18, 0.42)	5.24 (2.41, 11.39)	0.19 (0.09, 0.29)	3.32 (1.67, 6.60)
P for trend	0.000	0.000	0.000	0.000
P for trend, adjusted for real price index	0.000	0.000	0.000	0.000

Additional examination of the trend of daily smoking was carried out by age groups 25–44 and 45–64 years. For all men except for older less educated, the real price-adjusted trend of decreasing smoking prevalence was statistically significant (Online Resource B). Daily smoking among younger less educated men was more common than among older less educated men, but the differences decreased from 2009–2010 onwards. For women, smoking decreased among other groups (non-significant decrease among the highly educated older age group) but increased among the less educated older age group (real price index-adjusted odds ratio 2.89, 95% confidence interval 1.34–6.21) (Online Resource C). The differences in smoking between age groups among less educated women were notable at the beginning of the period but declined gradually to 2016.

## Discussion

Our 38-year follow-up of educational differences in smoking revealed that daily smoking decreased over time but was more common among men and the less educated during the whole study period. However, from the late 1970s, both absolute and relative differences in smoking between educational groups widened suggesting increasing inequalities in health in the future.

Trends for daily smoking seemed to be associated with the price of cigarettes, especially in the 2000s. A recent study including European countries proposed that lower socio-economic groups are more price sensitive (Hu et al. 2017). Our findings support this notion. Age-stratified examination showed that daily smoking declined over time as a general rule. Still, among the 45–64-year-old less educated women, smoking increased during the study period, possibly indicating a cohort effect (Helakorpi et al. 2008).

As Finland aims to be tobacco and nicotine free by 2030 (Finlex 2016), our results implicate that more attention should be especially taken concerning those in a lower socio-economic position. The support for smoking cessation should be enhanced, which along with large-scale campaigning, has been one of the weakest points of the Finnish tobacco control (Joossens and Raw 2017). As socio-economic differences in smoking cessation are observable in Finland (Bosdriesz et al. 2015), stop smoking services should be better targeted at lower socio-economic groups to reduce inequalities in health (Brown et al. 2014). Untargeted cessation services may reduce smoking altogether while still increasing inequalities in smoking (Brown et al. 2014).

The results support the general view that price is a strong instrument of tobacco control policy. The method of small gradual price increases has been used in Finland since 2009. The government has decided on a series of smaller consecutive tax increases which would gradually increase the average price of cigarettes altogether by 30% in 2016–2019. It has been estimated that long-term annual 10% increases in price would reduce socio-economic inequality in lung cancer mortality in England and in Wales (Soerjomataram et al. 2011). Price increases have also been considered to decrease inequalities in all-cause mortality in Finland (Kulik et al. 2013). Thus, further long-term price increases could be recommended together with national anti-tobacco campaigns with an emphasis on stopping smoking and help for quitting. This could lead to public discussion on tobacco by the media and could then also reach the less educated who are not easily reached by conventional methods. There is positive evidence from the past about a combined ‘shock effect’ of tobacco control measures (Pekurinen and Valtonen 1987), but it is important to be aware that sudden large tax increase may also backfire in terms of the illegal sales, for example.

Finland is at the final stage of the tobacco epidemic model, where the proportion of smokers and tobacco-related mortality is declining (Thun et al. 2012). However, the age-stratified examination revealed that there still are population groups with increasing smoking rates. We might see an increase in tobacco-related mortality among older less educated women in the future. This can be seen as part of the proposed fifth stage of the tobacco epidemic, where smoking among the lower socio-economic groups does not decrease (Dixon and Banwell 2009).

The role of tobacco control legislation in socio-economic differences in smoking has been studied, but its effect is inconclusive. Smoke-free workplace legislation in Finland has had a relatively largest effect on the decrease in smoking among industrial workers with less education (Heloma et al. 2001). Another study found the impact of the TCA less pronounced among male lower socio-economic groups in the early 2000s (Helakorpi et al. 2008). Educational differences in smoking have persisted or increased in Germany in the 2000s after implementing several tobacco control measures, such as smoke-free laws (Hoebel et al. 2018). In Switzerland, the implementation of a public smoking ban coincided with a widening of inequalities between socio-economic groups in 1995–2014 in terms of the smoking prevalence and quit ratio (Sandoval et al. 2018).

Point-of-sale ban decreased the smoking more among the less educated than among the highly educated in England (Kuipers et al. 2017). Pictorial warnings affect educational groups similarly (Brewer et al. 2016), but no studies on the impact of plain packaging have been published. One study suggests that pictorial health warning labels on plain packaging may affect smokers with higher socio-economic position more than smokers with lower socio-economic position (Swayampakala et al. 2017). Tobacco endgame could be seen as a strong measure to decrease and to eradicate inequalities in health (McDaniel et al. 2016). To our knowledge, no studies have examined the effects of the tobacco endgame as the target of the policy on socio-economic differences in smoking. The impact of the endgame and other novel tobacco control policy actions on inequalities in health should be monitored in the future.

Our results, in accordance with earlier studies (Hoebel et al. 2018; Lahelma et al. 2016; Hu et al. 2017), warrant further actions on reducing health inequalities. Even if smoking has declined among educational groups in Finland, socio-economic differences between these groups have widened. If the present trend with widening or relatively unchanged differences between educational groups continues and no new measures to change the trend are developed and implemented, it will predominantly be the less educated who are still smoking at the goal of the endgame in 2030.

### Strengths and limitations

Certain limitations need to be taken into account when interpreting our results. The declining response rate over time is a limitation. Earlier studies have shown that younger men, smokers, and the less educated are less likely to respond to surveys, and underreporting of smoking likely occurs (Reinikainen et al. 2018; Kopra et al. 2015). Observed differences in smoking between educational groups could have been even more pronounced had the less educated responded more actively. The number of observations in 2016 was lower compared with other study years, which may have influenced the power of statistical tests. Our results, especially from the last survey years, need to be interpreted with caution. The stratification of education according to tertiles could not always be determined exactly at 33% of the distribution.

This study has several strengths. The follow-up time is exceptionally long. Similar measures of education and smoking were used over the study period. The data were randomly sampled, and we used post-stratification weights in order to match the data distribution to the age–sex distribution of the general Finnish adult population. We were able to control the impact of the changes in the real price index on smoking.

### Conclusion

Since the late 1970s, smoking has decreased but differences between socio-economic groups have widened. More instruments for eradicating inequalities in health are needed, especially focusing on lower socio-economic groups. In addition to better support for smoking cessation and larger tax increases, other tobacco control policy actions should be considered. With even further actions, the objective of a tobacco and nicotine free Finland by 2030 may be attainable.

## Electronic supplementary material

Below is the link to the electronic supplementary material.

Supplementary material 1 (PDF 397 kb)
