# Supplementary material for: Thirty-eight-year trends of educational differences in smoking in Finland
Source: Int J Public Health. 2019 Mar 25;64(6):853–60. doi: 10.1007/s00038-019-01228-x (PMC6614163; doi:10.1007/s00038-019-01228-x)
Supplement: Supplementary file 1 — Supplementary material 1 (PDF 397 kb) [file 38_2019_1228_MOESM1_ESM.pdf]

Thirty-eight-year trends of educational differences in smoking in Finland. International Journal of Public Health.

Ruokolainen O[*], Heloma A, Jousilahti P, Lahti J, Pentala-Nikulainen O, Rahkonen O, Puska P.

*National Institute for Health and Welfare, Helsinki, Finland, otto.ruokolainen[at]thl.fi

Online Resource A Determination of smoking status variable. Health Behaviour and Health among the Finnish Adult Population / Regional Health and Well-being Study, 1978–2016.

Smoking status[a]	Have you ever smoked[b]	Have you ever smoked at least 100 times (cigarettes, cigars, pipes)?[c]	Have you ever smoked daily at least one year/how many years?[d]	When was the last time you smoked?[e]
1	.	.	.	1
1	.	.	2	1
1	2	.	.	1
1	2	2	.	1
1	2	2	2	1
2	.	.	1	.
2	2	.	1	.
2	2	2	.	2
2	2	2	1	1
2	2	2	1	2
2	2	2	2	2
3	2	2	.	3
3	2	2	2	3
3	2	2	2	4
4	.	.	.	7
4	2	.	.	5
4	2	.	.	7
4	2	2	.	5
4	2	2	.	6
4	2	2	.	7
4	2	2	2	5
4	2	2	2	6
4	2	2	2	7
5	.	1	.	.
5	1	.	.	.
5	2	.	1	3
5	2	.	1	4
5	2	.	1	5
5	2	.	1	7
5	2	1	.	.
5	2	2	1	3
5	2	2	1	4
5	2	2	1	5
5	2	2	1	6
5	2	2	1	7
9	2	.	.	.
9	2	2	.	.

Smoking status[a]	Have you ever smoked[b]	Have you ever smoked at least 100 times (cigarettes, cigars, pipes)?[c]	Have you ever smoked daily at least one year/how many years?[d]	When was the last time you smoked?[e]
9	2	2	1	.
9	2	2	2	.

. = No data

a: Smoking status values: 1= Daily smoker, 2= Occasional smoker, 3= Quitters 1–12 months ago, 4= Quitters over 1 year ago, 5= Never smoker, 9= Incomplete data.

Class 9 were omitted from further analyses and classes 3 and 4 were pooled to describe former smokers. Thus, the final smoking status variable consisted of four classes.

b: Values 1= No, 2= Yes

c: Values 1= No, 2= Yes

d: Values 1= No, 2= Yes

e: Values 1= Today or yesterday, 2= 2 days–1 month ago', 3= '1 month–six months ago', 4= 'six months–one year ago', 5= 'one year–five years ago', 6= 'six years–ten years ago', 7= 'over ten years ago'.

This classification have been used in reporting the Health Behaviour and Health among the Finnish Adult Population survey results http://urn.fi/URN:ISBN:978-952-302-447-2 .

Online Resource B Daily smoking men by education and age groups, age-adjusted. Finland, 1978–2016, Health Behaviour and Health among the Finnish Adult Population / Regional Health and Well-being Study

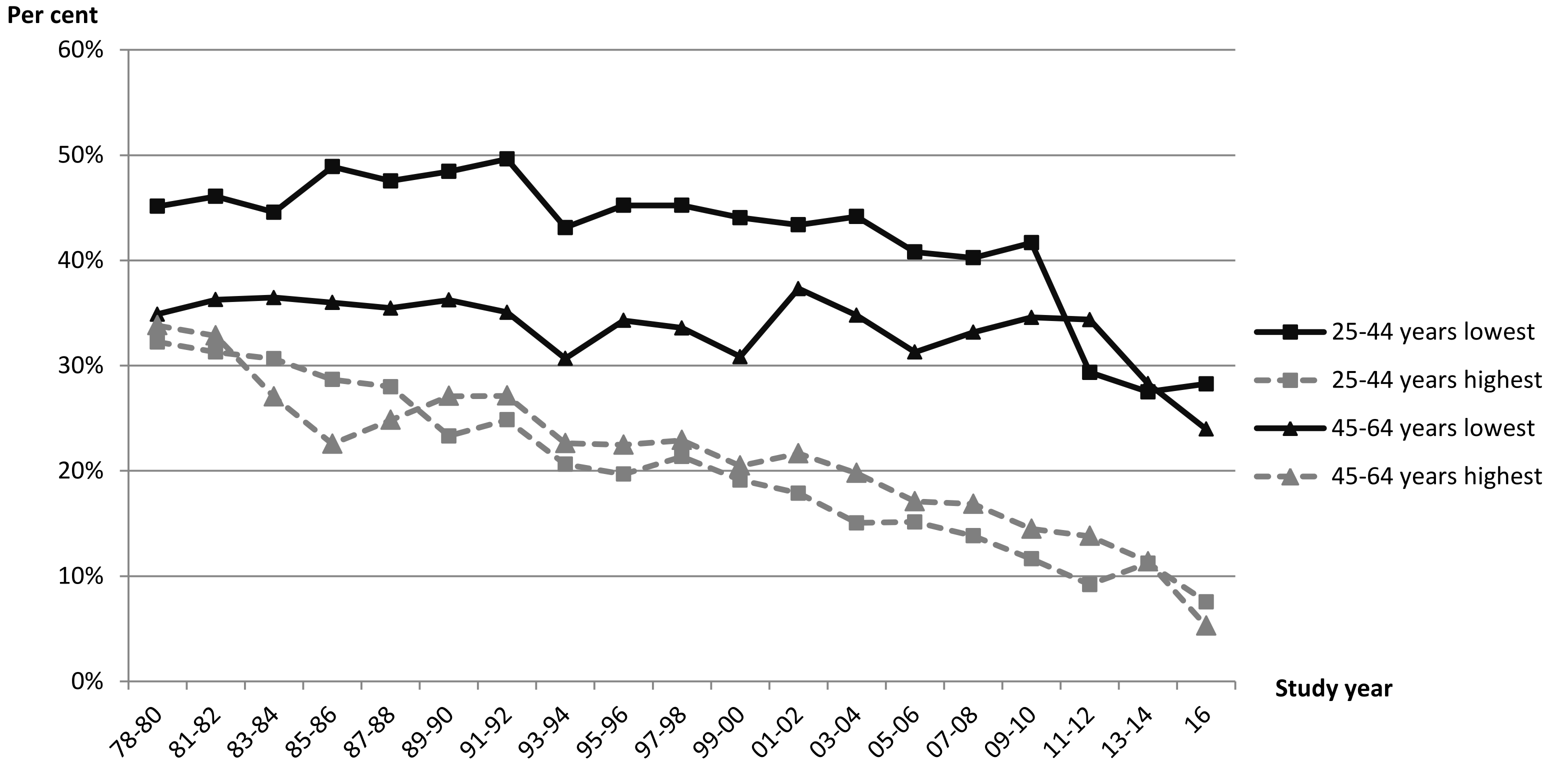

Men: Odds ratios and their 95% confidence intervals for trend over time in logistic regression model, daily smoking as outcome, continuous study year as independent variable**, adjusted for real price index**: 25–44 years lowest educated: 0.43 (0.22, 0.82), 25–44 years highest educated: 0.16 (0.07, 0.38), 45–64 lowest educated: 1.16 (0.58, 2.30), 45–64 highest educated: 0.20 (0.10, 0.43).

Men: Odds ratios and their 95% confidence intervals for trend over time in logistic regression model, daily smoking as outcome, continuous study year as independent variable: 25–44 years lowest educated: 0.59 (0.50, 0.69), 25–44 years highest educated: 0.20 (0.17, 0.25), 45–64 lowest educated: 0.81 (0.69, 0.96), 45–64 highest educated: 0.28 (0.24, 0.34).

Online Resource C Daily smoking women by education and age groups, age-adjusted. Finland, 1978–2016, Health Behaviour and Health among the Finnish Adult Population / Regional Health and Well-being Study

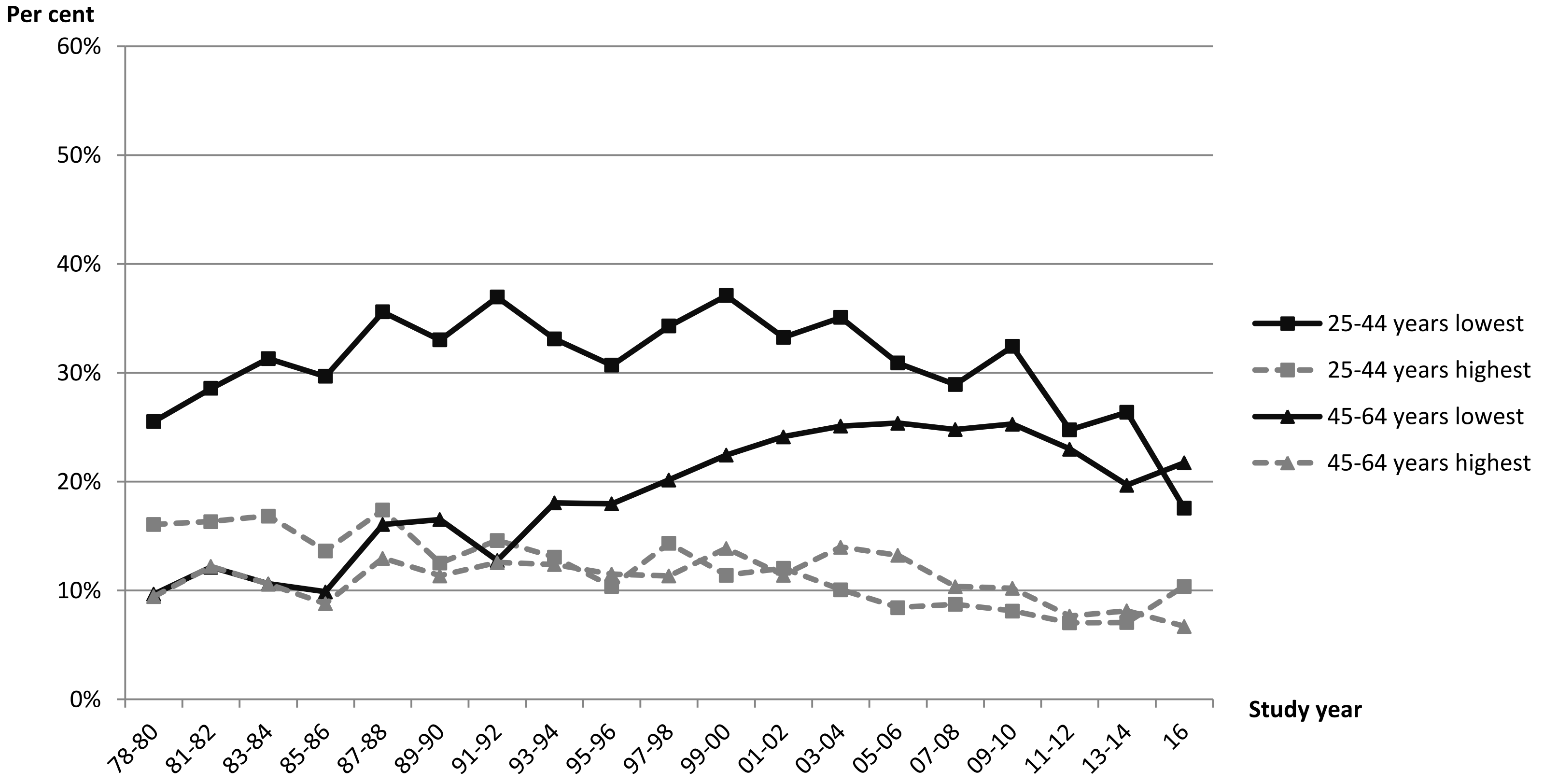

Women: Odds ratios and their 95% confidence intervals for trend over time in logistic regression model, daily smoking as outcome, continuous study year (and for lowest educated 25–45-year-olds its squared term) as the independent variable, **adjusted for the real price index**: 25–44 years lowest educated: 0.11 (0.05, 0.26), 25–44 years highest educated: 0.19 (0.08, 0.50), 45–64 lowest educated: 2.89 (1.34, 6.21), 45–64 highest educated: 0.55 (0.23, 1.35).

Women: Odds ratios and their 95% confidence intervals for trend over time in logistic regression model, daily smoking as outcome, continuous study year (and for lowest educated 25–44-year-olds its squared term) as the independent variable: 25–44 years lowest educated: 0.10 (0.05, 0.19), 25–44 years highest educated: 0.36 (0.29, 0.46), 45–64 lowest educated: 3.47 (2.87, 4.20), 45–64 highest educated: 0.91 (0.74, 1.13).
